# Emergency front-of-neck access in cardiac arrest: A scoping review

**DOI:** 10.1016/j.resplu.2024.100653

**Published:** 2024-05-04

**Authors:** Mohammed Aljanoubi, Abdulkarim A. Almazrua, Samantha Johnson, Ian R Drennan, Joshua C. Reynolds, Jasmeet Soar, Keith Couper, International Liaison Committee on Resuscitation Advanced Life Support Taskforce, Katherine M. Berg, Bernd W. Böttiger, Yew Woon Chia, Conor Crowley, Sonia D'Arrigo, Charles D. Deakin, Shannon M. Fernando, Rakesh Garg, Asger Granfeldt, Brian Grunau, Karen G. Hirsch, Mathias J. Holmberg, Eric Lavonas, Carrie Leong, Peter J. Kudenchuk, Peter Morley, Ari Moskowitz, Robert Neumar, Tonia C. Nicholson, Nikolaos Nikolaou, Jerry P. Nolan, Brian O'Neil, Shinichiro Ohshimo, Michael Parr, Helen Pocock, Claudio Sandroni, Tommaso Scquizzato, Markus Skrifvars, Neville Vlok, Michelle Welsford, Carolyn Zelop

**Affiliations:** a Warwick Clinical Trials Unit, University of Warwick, Coventry, UK; b Prince Sultan bin Abdul Aziz College for Emergency Medical Services, King Saud University, Riyadh, Saudi Arabia; c The Library, University of Warwick, Coventry, UK; d Division of Emergency Medicine, Department of Family and Community Medicine, University of Toronto, Toronto, Ontario, Canada; e Institute of Health Policy, Management and Evaluation, Dalla Lana School of Public Health, University of Toronto, Toronto, Ontario, Canada; f Department of Emergency Services and Sunnybrook Research Institute, Sunnybrook Health Science Centre, Toronto, Ontario, Canada; g Department of Emergency Medicine, Michigan State University, College of Human Medicine, Grand Rapids, MI, USA; h Southmead Hospital, North Bristol NHS Trust, Bristol, UK; i Critical Care Unit, University Hospitals Birmingham NHS Foundation Trust, Birmingham, UK

**Keywords:** Heart arrest, Out-of-hospital cardiac arrest, Emergency front-of-neck access, Endotracheal intubation, Cricothyroidotomy

## Abstract

**Background:**

Airway management is a core component of the treatment of cardiac arrest. Where a rescuer cannot establish a patent airway to provide oxygenation and ventilation using standard basic and advanced airway techniques, there may be a need to consider emergency front-of-neck airway access (eFONA, e.g., cricothyroidotomy), but there is limited evidence to inform this approach.

**Objectives:**

This scoping review aims to identify the evidence for the use of eFONA techniques in patients with cardiac arrest.

**Methods:**

In November 2023, we searched Medline, Embase, and Cochrane Central to identify studies on eFONA in adults. We included randomised controlled trials, non-randomised studies, and case series with at least five cases that described any use of eFONA. We extracted data, including study setting, population characteristics, intervention characteristics, and outcomes. Our analysis focused on four key areas: incidence of eFONA, eFONA success rates, clinical outcomes, and complications.

**Results:**

The search identified 21,565 papers, of which 18,934 remained after de-duplication. After screening, we included 69 studies (53 reported incidence, 40 reported success rate, 38 reported clinical outcomes; 36 studies reported complications). We identified only one randomised controlled trial. Across studies, there was a total of 4,457 eFONA attempts, with a median of 31 attempts (interquartile range 16–56.5) per study. There was marked heterogeneity across studies that precluded any pooling of data. There were no studies that included only patients in cardiac arrest.

**Conclusion:**

The available evidence for eFONA is extremely heterogeneous, with no studies specifically focusing on its use in adults with cardiac arrest.

## Introduction

Airway management is a core component of cardiac arrest management.^1^ During cardiac arrest, airway management by healthcare providers typically begins with bag-mask ventilation, followed by a stepwise escalation to more definitive strategies such as supraglottic airway insertion or tracheal intubation.^2^ Traditionally, tracheal intubation has been considered the gold standard for airway management in cardiac arrest, but out-of-hospital cardiac arrest randomised controlled trials of tracheal intubation during cardiopulmonary resuscitation during out-of-hospital cardiac arrest have shown that it is not superior to bag mask ventilation or supraglottic airway use.^3^, ^4^ There are ongoing studies on the role of tracheal intubation during CPR in-hospital cardiac arrest setting.^5^, ^6^

In some patients, standard basic and advanced airway techniques may not be effective leading to a cannot oxygenate scenario. The rescue strategy is the use of emergency front-of-neck access (eFONA) techniques, such as surgical cricothyroidotomy and needle cricothyroidotomy.^7^ A recent systematic review showed that pre-hospital use of eFONA had a very high success rate.^8^.

To date, the International Liaison Committee on Resuscitation (ILCOR) has not incorporated eFONA in its treatment recommendations for airway management in adult cardiac arrest. On this basis, the ILCOR Advanced Life Support (ALS) Task Force prioritised the need for a scoping review to explore the role of eFONA in adult cardiac arrest.

## Methods

The overarching objective of this scoping review was to explore the optimum airway management in cardiac arrest where initial strategies to achieve adequate ventilation and oxygenation have been unsuccessful, based on the following PICO (Population, Intervention, Comparator, Outcome) question:

In adult patients in cardiac arrest in any setting where adequate ventilation cannot be rapidly achieved using basic/advanced airway management strategies, does using a front-of-neck airway access attempt compared with ongoing attempts at basic/advanced airway management strategies change any clinical outcome?

We anticipated limited evidence in this specific area, so planned a scoping review that explored eFONA use in critically ill patients in the in-hospital and out-of-hospital settings, focussing on four key areas:

(1) Incidence of eFONA.
(2) Success rates of eFONA attempts.
(3) Clinical outcomes in patients with an eFONA attempt.
(4) Complications associated with eFONA attempts.

This scoping review was undertaken in line with ILCOR’s methodology for scoping reviews. This review paper is written in line with PRISMA checklist extension for scoping reviews.^9^ The ILCOR protocol template is available in the supplementary materials.

### Eligibility criteria

We included randomised controlled trials, non-randomised studies (e.g., interrupted time series, controlled before-and-after studies, and cohort studies), and case series with at least five patients that described any use of eFONA in the pre-hospital or in-hospital setting. We excluded paediatric studies where all patients aged < 18 years old, simulation studies, studies that describe non-emergency surgical airways, animal studies, case series/reports with fewer than five patients, editorials, protocols, review papers, and letters. Grey literature was eligible for inclusion. In studies that included adults and children, we extracted only adult data where possible.

### Information sources and search

We originally searched MEDLINE, Embase, and the Cochrane Library in June 2022, with search updates completed in January 2023 and November 2023. Search strategies were developed iteratively in collaboration with an information specialist (SJ). The searches combined keywords and index terms to describe the population, setting, and intervention of interest. The final search strategy is included in the supplementary materials. We did not limit the search by year of publication or language. We identified additional relevant studies, including grey literature, through liaison with subject experts and ILCOR Advanced Life Support Task Force members. We did not undertake specific searches for grey literature.

### Selection of sources of evidence

Following the completion of searches, the list of citations was deduplicated using Endnote X9 and X9.3.3, and Rayyan software (https://www.rayyan.ai/). Following deduplication, two reviewers (MA/AA) independently screened the titles and abstracts of the papers to exclude clearly irrelevant citations. The same two reviewers then independently reviewed the full text of potentially relevant studies. At each stage, reviewer conflicts were resolved through discussion or referral to a third reviewer.

### Data charting, items and critical appraisal

After developing and piloting a bespoke online form for data extraction, two reviewers (MA/AA) independently extracted data items, including study setting, population characteristics, intervention characteristics, and outcomes. An overview of extracted data items is included in the supplementary materials. Conflicts between reviewers were resolved through discussion or referral to a third reviewer. We did not undertake a critical appraisal of the evidence, as the primary focus of this scoping review was to describe the volume, type, and key findings of the available evidence.

### Synthesis of results

In line with the focus of this scoping review, we describe the findings in a narrative style for each of our four areas of focus. We present the results broken down for each key study setting (pre-hospital, in-hospital, pre-hospital/in-hospital, and battlefield) and summarise the findings of studies that focus specifically on cardiac arrest. Quantitative analysis consisted of descriptive analyses, including the frequencies and ranges of the outcomes for the included studies. The qualitative analysis comprised descriptions of the overall results and outcomes. For both methods, Microsoft Excel supported data analysis. Where appropriate, we calculated a 95% confidence interval for relevant outcomes in each study.

## Results

**Fig. 1:**
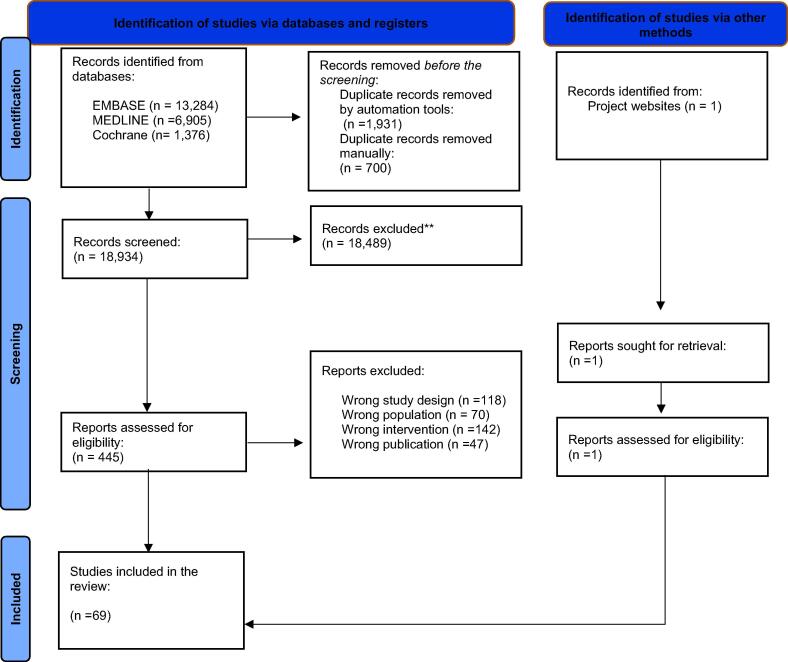

In total, our initial and updated searches identified 21,565 papers. Following the removal of duplicates, we screened 18,934 in the title/abstract stage, and then 445 full-text papers were retrieved for review (Fig. 1). In total, we included 69 studies.^10^, ^11^, ^12^, ^13^, ^14^, ^15^, ^16^, ^17^, ^18^, ^19^, ^20^, ^21^, ^22^, ^23^, ^24^, ^25^, ^26^, ^27^, ^28^, ^29^, ^30^, ^31^, ^32^, ^33^, ^34^, ^35^, ^36^, ^37^, ^38^, ^39^, ^40^, ^41^, ^42^, ^43^, ^44^, ^45^, ^46^, ^47^, ^48^, ^49^, ^50^, ^51^, ^52^, ^53^, ^54^, ^55^, ^56^, ^57^, ^58^, ^59^, ^60^, ^61^, ^62^, ^63^, ^64^, ^65^, ^66^, ^67^, ^68^, ^69^, ^70^, ^71^, ^72^, ^73^, ^74^, ^75^, ^76^, ^77^, ^78^ Of the 69 included studies, there was one randomised controlled trial and 68 observational studies. 10, 11, 12, 13, 14, 15, 16, 17, 18, 19, 20, 21, 22, 23, 24, 25, 26, 27, 28, 29, 30, 31, 32, 33, 34, 35, 36, 37, 38, 39, 40, 41, 42, 43, 44, 45, 46, 47, 48, 49, 50, 51, 52, 53, 54, 55, 56, 57, 58, 59, 60, 61, 62, 63, 64, 65, 66, 67, 68, 69, 70, 71, 72, 73, 74, 75, 76, 77, 78 The randomised controlled trial compared emergency cricothyrotomy and emergency percutaneous dilatational tracheotomy.^50^ Of the observational studies, 59 were retrospective, 7 were prospective, and 2 were case series. Forty-eight percent of the studies were conducted in the USA, and the rest were conducted in the UK, the Netherlands, Afghanistan, Iraq, Israel, Syria, Singapore, Germany, Denmark, Egypt, Korea, Japan, India, Australia and/or New Zealand.

Of the 69 studies, there were no studies that included only patients in cardiac arrest. In addition, 99% used surgical cricothyroidotomy as the eFONA technique, with or without using needle cricothyroidotomy or emergency tracheostomy in some cases. The included studies described a total of 4,457 eFONA attempts, with a median of 31 attempts (interquartile range 16–56.5) per study. Across studies, there was a range of reported rescuers, including emergency medicine physicians, anaesthetists, surgeons, paramedics, and nurses. Study duration ranged from several months to 20 years. Table 1 summarises the included studies.

**Table 1:** Characteristic of included studies.

Study (Date)	Design	Setting (Length)	Population	Intervention (Providers)†	Patients (Sex, Age)†	Reported Outcomes
Pre-hospital studies						
Spaite (1990)	RC	USA (1985–1987)	Trauma, CA	Surgical cric (ALS paramedics)	16	Success; clinical outcomes; complications
Boyle (1993)	RC	USA (1983–1988)	Trauma, CA	Surgical cric (air ambulance nurses)	69 (Sex: 78% M; Age: Mean 22 y)	Incidence; success rate; clinical outcomes complications
Xeropotamos (1993)	RC	UK (1991–1992)	Trauma, CA	Surgical cric (HEMS staff, physicians, surgeons)	11 (Age: Range 24–64 y)	Incidence eFONA; success rate; ROSC; clinical outcomes
Jacobson (1996)	RC	USA (1990–1994)	Trauma, CA	Surgical cric, needle cric (paramedics)	50 (Sex: 76% M; Age: Mean 32 y)	Incidence; success rate; ROSC; clinical outcomes; complications
Gerich (1998)	PC	Germany (1993–1997)	Trauma, Medical	Surgical cric (HEMS)	8 (Sex: 75% M; Age: Mean 40 y)	Incidence; success rate; clinical outcomes; complications
Thomas (1999)	RC	USA (1995–1997)	Trauma, Medical	Surgical cric (HEMS)	10	Incidence; success rate
Robinson (2001)	RC	USA (1985–1997)	Trauma, Medical	Surgical cric (nurses, physicians)	8	Incidence; success rate
Bulger (2002)	RC	USA (1997–1999)	Trauma, Medical, CA	Surgical cric, needle cric (paramedics)	30 (Sex: 70% M; Age: Range 18–99 y)	Incidence; clinical outcomes
Germann (2009)	RC	USA (1998–2006)	Trauma, CA	Surgical cric (paramedics, registered nurses)	6 (Sex: 100% M)	Incidence; success rate
Warner (2009)	PC	USA (2001–2005)	Trauma, CA	Surgical cric (paramedic, critical-care, advanced paramedics)	11	Incidence; success rate; clinical outcomes; complications
Wang (2011)	RC	USA (2008–2008)	Trauma, CA	Surgical cric, needle cric (standard paramedic physicians, surgeons)	88 (Sex: 75%M)	Incidence; success rate; complications
Shapey (2012)	RC	UK (2003–2010)	Trauma, Medical, CA	Surgical cric, needle cric (paramedics, doctors, HEMS)	16	Incidence; ROSC; complications
Kamiutsuri (2013)	RC	Japan (2004–2011)	Trauma, CA	Surgical cric (physicians)	13	Incidence; success rate
Brown (2014)	RC	USA (2007–2009)	Trauma, Medical	Surgical cric, needle cric (HEMS, paramedics, nurses)	35	Incidence; success rate
Prekker (2014)	RC	USA (2006–2011)	NA	Surgical cric, needle cric (paramedics)	30	Incidence; ROSC; clinical outcomes
Diggs (2014)	RC	USA (2012–2012)	NA	Surgical cric, needle cric (paramedics)	1,332	Incidence; success rate
Peters (2015)	RC	Netherlands (2007–2012)	NA	Surgical cric (HEMS physicians, paramedics, nurses)	19	Incidence
Peters (2015)	RC	Netherlands (2007–2013)	Trauma, Medical, CA	Surgical cric (anaesthesiologists, HEMS physicians, surgeons)	29 (Sex: 80% M)	Incidence; ROSC; clinical outcomes
Sunde (2015)	PC	Multicentre (2012–2013)	Trauma, Medical, CA	Surgical cric (HEMS physicians, paramedics)	6	Incidence
Gellerfors (2018)	RC	Multicentre (2015–2016)	Trauma, CA	Surgical cric (physicians, nurses)	14	Incidence
Schober (2019)	RC	Netherlands (2011–2018)	Trauma, Medical	Surgical cric, needle cric (HEMS)	18	Incidence; success rate; clinical outcomes; complications
Aziz (2021)	RC	UK (2000–2019)	Trauma, CA	Surgical cric, needle cric (physicians, paramedics)	72	Incidence; success rate
Himmler (2023)	RC	USA (2008–2020)	Medical, Surgery	Surgical cric	95	Incidence
Malkan (2023)	RC	USA (2007–2020)	Trauma	Surgical cric	251 (Sex: 98% M; Age: Median 25 y)	Incidence; clinical outcomes; complications
In-hospital setting						
McGill (1982)	RC	USA (1977–1980)	Trauma, Medical, CA	Surgical cric (ER physicians, surgeons)	38 (Age: Average 41 y)	Incidence; clinical outcomes;
Erlandson (1989)	RC	USA (1981–1985)	Trauma, Medical, CA	Surgical cric (ER physicians, anaesthesiologists, surgeons)	39	Incidence; clinical outcomes; complications
Delaurier (1990)	RC	USA (1984–1988)	Trauma	Surgical cric (ER physicians)	34	Clinical outcomes; complications
Gillespie (1999)	RC	USA (1993–1998)	Trauma, Medical, CA	Surgical cric, needle cric, tracheostomy (ER physicians, surgeons)	35 (Sex: 69% M; Age: Mean 50 y)	Success rate; complications
Isaacs (2001)	RC	USA (1996)	Trauma, Medical	Surgical cric	27 (Sex: 70% M; Age: Range 20–81 y)	Clinical outcomes; complications
Bair (2002)	RC	USA (1998–2001)	Trauma, Medical	Surgical cric, needle cric, tracheostomy (ER physicians surgeons)	44	Incidence
Wong (2008)	PC	Singapore (2000–2006)	Trauma, Medical, CA	Surgical cric, tracheostomy (ER physicians, anaesthesiologists, Surgeons)	7	Incidence; success rate
Cook (2011)	RC	UK (2008–2009)	Trauma, Medical, CA	Surgical cric, needle cric, tracheostomy (ER physicians, anaesthesiologists, surgeons)	75 (Sex: 58% M)	Incidence; success rate; complications
NAP4 (2011)	PC	UK (2008–2009)	Trauma, Medical, CA	Surgical cric, needle cric (ER physicians, anaesthesiologists, surgeons)	58	Incidence; success rate clinical outcomes; complications
Beshey (2014)	RCT	Egypt (2011–2011)	Trauma, Medical, CA	Surgical cric	169 (Percutaneous cricothyroidotomy = 85 and Percutaneous dilational tracheostomy = 84); (Age: Mean 46 ± 32 y)	Incidence; success rate; complications
Rosenstock (2016)	RC	Denmark (2008–2014)	Trauma, Medical, CA	Surgical cric (anaesthesiologists, surgeons)	27 (Sex: 78% M; Age: Mean 57 y)	Incidence; success rate; complications
Darby (2018)	RC	USA (2008–2012)	Trauma, Medical, CA	Surgical cric, needle cric (physicians, anaesthesiologists, surgeons)	22 (Sex: 77% M; Age: Mean 61 ± 11)	Incidence; success rate; Clinical outcomes; Complications
Kwon (2019)	RC	Korea (2007–2018)	Trauma, Medical, CA	Surgical cric, needle cric (ER, ENT)	23 (Sex: 78% M; Age: Mean 63 y)	Success rates; ROSC; clinical outcomes; complications
Alkhouri (2020)	RC	Australia, New Zealand (2010–2015)	Trauma, Medical, CA	Surgical cric, tracheostomy (ER-physicians, intensivists, anaesthetists, GP)	15 (Sex: 93% M; Age: Mean 54 y)	Complications
Willinge (2021)	RC	Netherlands (2013–2018)	Trauma, Medical, CA	Surgical cric (surgeons)	52 (Age: Median 54 y)	Complications
Okada (2022)	RC	Japan (2012–2020)	Trauma, Medical, CA	Surgical cric, needle cric, tracheostomy (physicians)	31 (Sex: 74% M; Age: Median 53 y)	Incidence; clinical outcomes; complications
George (2022)	RC	USA (2009–2019)	Trauma	Surgical cric	51 (Sex: 77% M; Age: Average 45 ± 19 y)	Incidence; clinical outcomes
Jansen (2023)	RC	Germany (2014–2019)	In-hospital emergencies	Surgical cric	8	Incidence
Arora (2023)	RC	India (2021–2022)	Medical	Needle cric, tracheostomy	17 (Sex: 41% M; Age: Mean 64 y)	Clinical outcomes
Offenbacher (2023)	RC	USA (2016–2018)	Trauma, Medical, CA	Surgical cric	49 (Sex:80% M; Age: Median 41 y)	Incidence; success rate; clinical outcomes
In-hospital/pre-hospital settings						
Nugent (1991)	RC	USA (1987–1989)	Trauma, Medical, CA	Surgical cric (HEMS-nurses)	55 (Sex: 78% M; Age: Range 9–76 y)	Incidence; success rate; clinical outcomes; complications
Salvino (1993)	RC	USA (1988–1991)	Trauma	Surgical cric (paramedics, HEMS-nurses and paramedics, anaesthesiologists, surgeons)	30 (Sex: 90% M)	Incidence; success rate; clinical outcomes; complications
Hawkins (1995)	RC	USA (1989–1993)	Trauma	Surgical cric(ER-physicians)	66	Incidence; clinical outcomes; complications
Bair (2003)	RC	USA (1995–2000)	Trauma, Medical	Surgical cric (HEMS, ER-physicians, surgeons)	50	Incidence; success rate; complications
McIntosh (2008)	RC	USA (1995–2004)	Trauma, Medical	Surgical cric (paramedics, HEMS-nurses, and paramedics)	17	Incidence; success rate clinical outcomes; complications
Graham (2011)	RC	USA (1995–2010)	Trauma, Medical, CA	Surgical cric	94 (Sex: 94% M)	Success rate; ROSC; clinical outcomes; complications
Paix (2012)	Case series	Australia (1992–2011)	Trauma, Medical, CA	Surgical cric, needle cric	24 (Sex: 88% M)	Success rate; complication
King (2012)	RC	USA (2000–2010)	Trauma	Surgical cric (paramedics, ER-physicians, surgeons)	54 (Sex: 80% M; Age: Mean 50 ± 15)	Complications
Katzenell (2013)	RC	Israel (1997–2010)	Trauma	Surgical cric (paramedics, physicians)	46	Incidence; success rate; clinical outcomes.
High (2018)	RC	USA (2006–2015)	Trauma, Medical	Surgical cric (HEMS)	13 (Sex: 75% M)	Incidence; success rate
Duggan (2018)	RC	Multicentre (2016–2017)	Trauma, Medical, CA	Surgical cric, needle cric, tracheostomy (paramedics; ER-physicians, anaesthesiologists, surgeons)	99 (Sex: 74% M)	Success rate
Morocco (2021)	Case series	USA (2010–2020)	Trauma	Surgical cric (paramedics, surgeons)	12 (Sex: 92% M; Age: Average 43 y)	Incidence; success rate; clinical outcomes; complications
Battlefield						
Leibovici (1997)	RC	Israel (1991–1995)	Trauma	Surgical cric	29 (Age: Median 20 ± 6 y)	Success rate; clinical outcomes; complications
Adams (2008)	PC	Iraq (2005–2007)	Trauma, Medical, CA	Surgical cric	17 (Sex: 95% M)	Incidence; success rate; complications
Mabry (2012)	RC	Iraq, Afghanistan (2007–2009)	Trauma	Surgical cric	72 (Sex: 96% M)	Success rate; clinical outcomes; complications
Lairet (2012)	PC	Afghanistan (2009–2011)	NA	Surgical cric	15	Incidence; complications
Barnard (2014)	RC	Afghanistan (2009–2013)	Trauma	Surgical cric (ground and flight-medics)	34 (Sex: 97% M; Age: median 24y)	Incidence; success rate; clinical outcomes
Pugh (2015)	RC	Afghanistan (2013–2013)	Trauma	Surgical cric (paramedics)	14	Incidence; clinical outcomes; complications
Schauer (2015)*	RC	USA (2010–2012)	Trauma	Surgical cric (military-physicians and physician-assistants)	32 (Age: Range ]36–56 y [)	Incidence; clinical outcomes
Tobin (2015)	RC	Afghanistan (2010–2010)	Trauma, CA	Surgical cric, tracheostomy (military personnel, HEMS- critical care team).	42	Incidence
Kyle (2016)	RC	Afghanistan (2006–2014)	Trauma	Surgical cric (general medic, medical emergency response team, combat EMT)	86 (Sex: 100% M; Age: Median 25 y)	Success rate; clinical outcomes
Schauer (2018)	RC	Iraq, Afghanistan (2007–2016)	Trauma	Surgical cric	230 (Afghanistan = 178 and Iraq = 52); (Sex: Iraq: 96% M; Afghanistan: 99% M)	Clinical outcomes; complications
Benov (2019)	RC	Israel, Syria (2013–2017)	Trauma, Medical	Surgical cric	30 (Male:93%; Age: Median 24 y)	Incidence; success rate
Hudson (2020)	RC	Afghanistan (2008–2014)	Trauma	Surgical cric, tracheostomy	85 (Sex: 98% M; Age: Median 25 y IQR [23–30])	Incidence; clinical outcomes.
Beit ner (2021)	RC	Israel (1998–2018)	Trauma	Surgical cric (paramedics, ER-physicians)	153 (Age: Mean 27 y)	Incidence; success rate; clinical outcomes

Key: ALS – Advanced Life Support, CA – cardiac arrest, Cric – cricothyroidotomy, EMT – Emergency medical technician, ENT – Ear-Nose-Throat surgeon, ER – emergency room, GP – General practitioner (primary care doctor), HEMS – Helicopter Emergency Medical Services, HRQoL – Health-related quality of life), M – Male, PC – prospective cohort RC – retrospective cohort-Year.

† Provider/Age/sex information reported where available.

### Incidence of eFONA

The incidence of eFONA was described in 53 studies.^42^, ^45^, ^46^, ^54^, ^65^, ^69^, ^72^, ^10^, ^11^, ^12^, ^13^, ^14^, ^16^, ^17^, ^18^, ^19^, ^20^, ^21^, ^22^, ^23^, ^24^, ^25^, ^26^, ^27^, ^28^, ^29^, ^30^, ^31^, ^32^, ^33^, ^34^, ^36^, ^37^, ^38^, ^39^, ^40^, ^48^, ^49^, ^50^, ^51^, ^56^, ^57^, ^58^, ^60^, ^61^, ^62^, ^74^, ^75^, ^76^, ^77^, ^78^ Studies were conducted across the pre-hospital setting (*n* = 23, 43%), in-hospital setting (*n* = 13, 25%), pre-hospital/in-hospital setting (*n* = 8, 15%), and on the battlefield (*n* = 9, 17%). There were important differences in the denominator used to calculate incidence across studies, including all EMS calls, patients in whom tracheal intubation was attempted, and undefined population types (Table 2). None of the studies reported an incidence rate for patients in cardiac arrest. The lowest reported incidence was 0.06 per 1,000 patients receiving general anaesthesia, and the highest incidence was 436 per 1,000 patients with an identified difficult airway.^36^, ^37^

**Table 2:** Incidence rate of eFONA.

Study	eFONA attempts	Number in population (population definition)	Incidence per1000 (95% CI)
Pre-hospital			
Boyle (1993)	69	2,188 (air ambulance calls)	31.53 (24.61–39.74)
Xeropotamos (1993)	11	600 (treated by HEMS)	18.33 (9.18–32.56)
Jacobson (1996)	50	14,772 (transported by ambulance services)	3.38 (2.51–4.46)
Gerich (1998)	8	383 (airway management required)	20.88 (9.05–40.74)
Thomas (1999)	10	722 (airway management attempts)	13.85 (6.66–25.32)
Robinson (2001)	8	1,589 (tracheal intubation required)	11.47 (7.75–16.34)
Bulger (2002)	30	2,614 (tracheal intubation attempts)	8.41 (5.28–12.71)
Germann (2009)	6	369 (tracheal intubation attempts)	16.26 (5.99–35.05)
Warner (2009)	11	4,091 (tracheal intubation attempts)	2.68 (1.34–4.80)
Wang (2011)	88	88,180 (airway management attempts)	0.99 (0.80–1.22)
Shapey (2012)	16	5,490 (EMS calls)	2.91 (1.66–4.72)
Kamiutsuri (2013)	13	3,719 (treated by EMS)	3.49 (1.86–5.97)
Brown (2014)	35	4,871 (tracheal intubation attempts)	7.18 (5.01–9.97)
Prekker (2014)	30	7,523 (advanced airway attempts)	3.98 (2.69–5.68)
Diggs (2014)	1332	136,980 (airway management attempts)	9.72 (9.21–10.25)
Peters (2015)	19	1,399 (airway management required)	13.58 (8.19–21.12)
Peters (2015)	29	1,871 (airway management required)	15.49 (10.40–22.18)
Sunde (2015)	6	2,327 (tracheal intubation required)	2.57 (0.94–5.60)
Gellerfors (2018)	9	2,054 (tracheal intubation required)	4.38 (2.01–8.30)
Schober (2019)	18	10,252 (air ambulance Calls)	1.75 (1.04–2.77)
Aziz (2021)	72	37,725 (EMS calls)	1.90 (1.49–2.40)
Himmler (2023)	95	953 (critical airway team activations)	99.68(81.40–120.49)
Malkan (2023)	251	258,976 (cases registered)	0.96 (0.85–1.09)
In-hospital			
McGill (1982)	38	1,362 (tracheal intubation required)	27.90 (19.81–38.09)
Erlandson (1989)	39	2,287 (tracheal intubation required)	17.05 (12.15–23.23)
Bair (2002)	22	7,712 (tracheal intubation attempts)	2.85 (1.78–4.31)
Wong (2008)	8	2,343 (advanced airway required)	3.41 (1.47–6.72)
NAP4 (2011)	58	133 (difficult airway cases)	436.09 (350.34–524.74)
Cook (2011)	75	286 (major complications of airway management reports)	262.23 (212.21–317.26)
Beshey (2014)	163	3,785 (advanced airway required)	43.06 (36.82–50.03)
Rosenstock (2016)	27	452,461 (general anaesthesia patients)	0.06 (0.03–0.08)
Darby (2016)	22	266 (difficult airway cases)	82.70 (52.55–122.54)
George (2022)	51	29,213 (tracheal intubation required)	1.75 (1.30–2.29)
Okada (2022)	31	75,529 (emergency cases)	0.41 (0.27–0.58)
Jansen (2023)	8	14,166(emergency interventions)	0.56 (0.24–1.11)
Offenbacher (2023)	49	17,720 (tracheal intubation attempts)	2.76 (2.05–3.65)
Pre-hospital /in-hospital			
Nugent (1991)	55	302 (airway management required)	185.43 (143.21–233.93)
Salvino (1993)	30	8,320 (trauma admissions)	3.61 (2.43–5.14)
Hawkins (1993)	66	525 (airway management required)	125.71 (98.57–157.14)
Bair (2003)	50	2,730 (tracheal intubation attempts)	18.31 (13.62–24.07)
McIntosh (2008)	17	712 (advanced airway required)	23.87 (13.96–37.95)
Katzenell (2012)	46	406 (tracheal intubation attempts)	113.31 (84.15–148.22)
High (2018)	13	22,434 (EMS calls)	0.57 (0.31–0.99)
Moroco (2021)	12	1,642 (trauma cases identified)	7.30 (3.78–12.73)
Battlefield			
Adams (2008)	17	293 (advanced airway attempts)	58.02 (34.15–91.27)
Lairet (2012)	15	1,003 (combat cases)	14.95 (8.39–24.54)
Barnard (2014)	34	1,927 (cases identified)	17.64 (12.24–24.56)
Pugh (2015)	14	57 (advanced airway attempts)	245.61 (141.26–377.61)
Schauer (2015)	32	14,233 (trauma admissions)	2.24 (1.53–3.17)
Tobin (2015)	42	1,198 (transportation events)	35.05 (25.38–47.09)
Benov (2019)	30	134 (advanced airway attempts)	223.88 (156.43–303.92)
Hudson (2020)	85	890 (airway management attempts)	95.51 (76.99–116.74)
Beit Ner (2021)	153	17,702 (recorded casualties)	8.64 (7.33–10.11)


EMS: Emergency medical services; HEMS: Helicopter emergency medical services.

### eFONA success rates

The eFONA success rate was reported in 40 studies.^15^, ^17^, ^18^, ^23^, ^24^, ^26^, ^27^, ^29^, ^36^, ^37^, ^41^, ^44^, ^60^, ^61^, ^70^, ^71^, ^73^, ^10^, ^11^, ^12^, ^13^, ^31^, ^32^, ^33^, ^34^, ^48^, ^49^, ^50^, ^54^, ^55^, ^56^, ^57^, ^58^, ^63^, ^64^, ^65^, ^75^, ^76^, ^77^, ^78^ The most common location was the pre-hospital setting (*n* = 15, 38%), followed by in-hospital setting (*n* = 8, 20%), pre-hospital/in-hospital setting (*n* = 10, 25%) and in the battlefield (*n* = 7, 18%). Six studies reported a success rate of less than 70%.^12^, ^18^, ^29^, ^49^, ^57^, ^70^ However, 22% of the studies reported a 100% success rate of the performed eFONA.^10^, ^27^, ^54^, ^58^, ^61^, ^63^, ^65^ The median success rate of eFONA among all settings was 91% (Table 3). None of the studies reported a success rate for patients in cardiac arrest.

**Table 3:** Efona success rate.

Study	Number of eFONA cases	Number of successful eFONA	Success rate% (95% CI)
Pre-hospital			
Spaite (1990)	16	14	88% (62–98)
Boyle (1993)	69	68	99% (92–99)
Xeropotamos (1993)	11	11	100% (71–100*)
Jacobson (1996)	50	47	94% (83–98)
Gerich (1998)	8	8	100% (63–100*)
Thomas (1999)	10	9	90% (55–99)
Robinson (2001)	8	5	63% (24–91)
Warner (2009)	10	9	90% (55–99)
Germann (2009)	6	6	100% (54–100*)
Wang (2011)	88	61	69% (58–78)
Kamiutsuri (2013)	13	11	85% (54–98)
Diggs (2014)	1332	457	34% (31–36)
Brown (2014)	35	34	97% (85–99)
Schober (2019)	230	216	94% (89–96)
Aziz (2021)	11	10	91% (58–99)
In-hospital			
Gillespie (1999)	35	34	97% (85–99)
Wong (2008)	8	7	88% (47–99)
Cook (2011)	58	21	36% (23–49)
NAP4 (2011)	58	50	86% (74–93)
Beshey (2014)	169	163	96% (92–98)
Darby (2016)	22	20	91% (70–98)
Rosenstock (2016)	27	21	78% (57–91)
Kwon (2019)	23	17	74% (51–89)
Pre-hospital/In-hospital			
Nugent (1991)	55	53	96% (87–99)
Salvino (1993)	30	30	100% (88–100*)
Bair (2003)	50	50	100% (92–100*)
McIntosh (2008)	17	17	100% (80–100*)
Graham (2011)	94	94	100% (96–100*)
Paix (2012)	24	24	100% (85–100*)
Katzenell (2012)	46	43	93% (82–98)
Duggan (2018)	99	71	72% (61–80)
High (2018)	13	13	100% (75–100*)
Moroco (2021)	12	7	58% (27–84)
Battlefield			
Leibovici (1997)	29	26	90% (72–97)
Adams (2008)	17	13	76% (50–93)
Mabry (2012)	72	49	68% (56–78)
Barnard (2014)	34	28	82% (65–93)
Kyle (2016)	86	79	92% (83–96)
Benov (2019)	30	25	83% (65–94)
Beit Ner (2021)	153	135	88% (82–92)

* One-sided, 97.5% confidence interval.

### Clinical outcomes

Clinical outcomes in patients who received eFONA were reported in 38 studies.^10^, ^11^, ^15^, ^17^, ^19^, ^21^, ^22^, ^24^, ^27^, ^30^, ^32^, ^36^, ^43^, ^52^, ^54^, ^60^, ^62^, ^63^, ^73^, ^74^, ^76^, ^39^, ^40^, ^41^, ^45^, ^46^, ^47^, ^48^, ^56^, ^57^, ^58^, ^67^, ^68^, ^69^, ^70^, ^71^ Of these, 11 (29%) were pre-hospital studies, 11 (29%) were in-hospital studies, seven (18%) were in-hospital/pre-hospital, and nine (24%) were battlefield studies.

Nine studies reported the rate of return of spontaneous circulation (ROSC) in patients who had a cardiac arrest and who received eFONA at some point during their treatment.^10^, ^16^, ^18^, ^19^, ^21^, ^24^, ^32^, ^41^, ^63^ Across studies, the return of spontaneous circulation rate ranged from 0% to 64% (Table 4).

**Table 4:** Summary of outcomes in patients with eFONA that sustained a cardiac arrest.

Setting	Number of cardiac arrest patients (number of studies) Setting	Return of spontaneous circulation
Pre-hospital	112 (Seven studies)^10^, ^16^, ^18^, ^19^, ^21^, ^24^, ^32^	20% (range 0–64%)
In-hospital	17 (One study)^41^	41%
Pre-hospital and in-hospital	47 (One study)^63^	0%
Battlefield	NA	NA

Eleven studies (seven pre-hospital, one in-hospital, and three battlefield) reported the outcome of survival to hospital admission.^11^, ^17^, ^19^, ^21^, ^27^, ^30^, ^32^, ^39^, ^69^, ^71^, ^74^ Across studies, the survival rate of hospital admission ranged from 19% to 73% (Table 5).

**Table 5:** Clinical outcomes across all patients with eFONA.

Setting	Number of patients (number of studies)	Clinical outcome
Survival to hospital admission		
Pre-hospital	195 (seven studies)^11^, ^17^, ^19^, ^21^, ^27^, ^30^, ^32^	29% (range 19–91%)
In-hospital	49 (One study)^39^	73%
Pre-hospital and in-hospital	NA	NA
Battlefield	127 (Three studies)^69^, ^71^, ^74^	53% (range 46–55%)
Survival to hospital discharge/30-days		
Pre-hospital	423(Seven studies)^10^, ^15^, ^17^, ^22^, ^24^, ^27^, ^32^	64% (range 7–98%)
In-hospital	255 (Eight studies)^38^, ^40^, ^41^, ^46^, ^47^, ^48^	42% (range 13–82%)
Pre-hospital and in-hospital	227 (Six studies) 54, 60, 57, 58	32% (range 27–75%)
Battlefield	692 (Seven studies)^67^, ^68^, ^70^, ^73^, ^74^, ^76^, ^77^	45% (range 4–67%)
Survival to hospital discharge/30-days with good functional outcome		
Pre-hospital	77 (Three studies)^10^, ^15^, ^24^	5% (range 0–27%)
In-hospital	158 (Four studies)^36^, ^43^, ^46^, ^47^	44% (range 5–69%)
Pre-hospital and in-hospital	112 (Two studies)^58^, ^63^	33% (range 29–34%)
Battlefield	34 (One study)^77^	9%

Twenty-eight studies (seven pre-hospital, eight in-hospital, six pre-hospital/in-hospital, and seven battlefield) reported the outcome of survival until hospital discharge or 30-days. 10, 15, 17, 22, 24, 27, 32, 38, 40, 41, 52, 54, 60, 62, 67, 68, 70, 73, 74, 76, 46, 47, 48, 56, 57, 58 In these studies, survival ranged from 4 to 98%. Most studies reported a survival rate of less than 67% (Table 5).

Ten studies (three pre-hospital, four in-hospital, two pre-hospital/in-hospital, and one battlefield) reported survival to hospital discharge or 30 days with favourable neurological outcomes.^10^, ^15^, ^24^, ^36^, ^43^, ^46^, ^47^, ^58^, ^63^, ^77^ The survival to hospital discharge or 30 days with favourable neurological outcome ranged from 0% to 69%. One study used the Glasgow Coma Scale (GCS) to assess neurological outcomes (Table 5).^63^.

Five studies (three pre-hospital and two in-hospital setting studies) reported health-related quality-of-life outcomes. None of the studies specified which scales or parameters were used to measure the results. Two studies reported that patients could speak normally after discharge.^40^, ^46^ Poor patient health after eFONA was reported in one study, but another reported good recovery after the procedure.^10,^ Finally, one study reported that patients could care for themselves and were in an ambulatory status.^32^

### Complications

Overall, 36 studies (eight (22%) pre-hospital, 13 (36%) in-hospital, nine (25%) combined in-hospital and pre-hospital, six (17%) in the battlefield setting) reported complications with eFONA.^11^, ^12^, ^20^, ^22^, ^24^, ^27^, ^32^, ^40^, ^41^, ^43^, ^44^, ^53^, ^54^, ^62^, ^63^, ^65^, ^67^, ^70^, ^71^, ^16^, ^17^, ^35^, ^36^, ^37^, ^38^, ^46^, ^47^, ^48^, ^49^, ^50^, ^56^, ^57^, ^58^, ^59^ Four studies reported immediate complications, including incorrect site of tube placement, procedure time greater than 3 min, and haemorrhage.^32^, ^40^, ^56^, ^63^ One study reported a long-term complication: laryngeal fracture with permanent dysphonia.^40^ Other studies reported a range of other complications, such as bleeding, tracheal erosion and supraglottic inflammation.

## Discussion

Our scoping review, which included total of 4,457 eFONA attempts across 69 studies, provides important information on eFONA incidence, success rates, clinical outcomes, and complications. We identified no studies specific to adults in cardiac arrest. We found marked variation in the denominator used to calculate eFONA incidence with associated marked variation in incidence across studies. The reported success rates of eFONA were generally high and clinical outcomes appear to be influenced by the study setting.

Our scoping review builds on Morton and colleague’s recently published systematic review and meta-analysis that focused on success rates of eFONA in the pre-hospital setting.^8^ Morton et al.’s systematic review included 69 studies and found an overall eFONA success rate of 88%, with higher success rates reported with surgical techniques (92%) than needle techniques (52%). In contrast to that review, our review had a broader scope and we chose to exclude small case series due to their high risk of selection bias, such that we included fewer pre-hospital studies. Nevertheless, the reported overall success rate in our review (91%) was comparable to that reported by Morton et al (88%).

The European Resuscitation Council and American Heart Association both recognise that there may be a need for eFONA during cardiac arrest.^1^, ^79^ There are likely two main patient groups in which eFONA may need to be considered. First, a patient might have a hypoxic cardiac arrest in an established “cannot intubate, cannot oxygenate” scenario, where face-mask ventilation and placement of a supraglottic airway device has already been unsuccessful. In this case, UK Difficult Airway Society guidelines provide a clear framework for progression to eFONA as a ‘Plan D’ airway management strategy.^7^

The second patient group is patients in cardiac arrest in which providers are unable to successfully site a tracheal tube or supraglottic airway, due to patient anatomy, cardiac arrest aetiology, or environmental factors. In such cases, UK Difficult Airway Society guidelines recommend that providers attempt face-mask ventilation.^7^ However, the adequacy of face-mask ventilation can be challenging to assess during cardiac arrest due to ongoing chest compressions and because standard strategies for determining adequate oxygenation, such as pulse oximetry, are unreliable in cardiac arrest.^1^, ^80^ In such cases, healthcare providers will need to make a clinical judgement regarding the need for eFONA. Recent qualitative research with UK critical care paramedics described the stress associated with making a decision to perform eFONA.^81^ Our review findings provide reassurance that when appropriately skilled healthcare providers attempt eFONA, the success rate is usually high in all clinical settings, although index studies rarely provided information on provider training or exposure to eFONA. There is a need for further studies that report the incidence, success rate, and outcomes of patients in cardiac arrest that receive eFONA. However, undertaking high-quality studies is likely to be extremely challenging due to the low incidence of eFONA in this population.

This review has two important limitations. First, the index studies were extremely heterogeneous, in relation to study design, healthcare provider, and setting. Second, we chose not to undertake a risk or bias assessment as our overriding objective was to characterise the volume and type of existing evidence, rather than draw conclusions to inform clinical practice.^82^ Third, we chose to focus our scoping review on eFONA in adults. eFONA placement in children may be more challenging due to both patient factors and provider confidence and expertise, limiting the generalisability of our findings to children.^83^ A recent review highlighted that few data are available on eFONA in children.^83^

## Conclusion

Our scoping review found no studies specifically focusing on the use of eFONA during adult cardiac arrest. The available evidence suggests that the incidence of eFONA is generally low, but that, when attempted, the success rate is typically high.

## Funding

MA and AAA are undertaking PhDs which are financially supported by Prince Sultan College for Emergency Medical Services, King Saud University, Riyadh, Saudi Arabia.

## CRediT authorship contribution statement

**Mohammed Aljanoubi:** Writing – review & editing, Writing – original draft, Methodology, Formal analysis, Conceptualization. **Abdulkarim A. Almazrua:** Writing – review & editing, Investigation. **Samantha Johnson:** Writing – review & editing, Investigation. **Ian R Drennan:** Writing – review & editing, Investigation, Conceptualization. **Joshua C. Reynolds:** Writing – review & editing, Investigation, Conceptualization. **Jasmeet Soar:** Writing – review & editing, Investigation, Conceptualization. **Keith Couper:** Writing – review & editing, Writing – original draft, Supervision, Methodology, Conceptualization.

## Declaration of competing interest

The authors declare the following financial interests/personal relationships which may be considered as potential competing interests: ‘JS is an Editor of the journal Resuscitation (payment received from the publisher, Elsevier) and Editorial Board member of Resuscitation Plus. KC is an Associate Editor of Resuscitation Plus (payment received from the publisher, Elsevier). IRD, JCR, JS, and KC are current or former members of the ILCOR ALS task force.’.

## References

[1] Soar J., Böttiger B.W., Carli P. (2021). European resuscitation council guidelines 2021: adult advanced life support. Resuscitation.

[2] Newell C., Grier S., Soar J. (2018). Airway and ventilation management during cardiopulmonary resuscitation and after successful resuscitation. Critical Care.

[3] Benger J.R., Kirby K., Black S. (2018). Effect of a strategy of a supraglottic airway device vs tracheal intubation during out-of-hospital cardiac arrest on functional outcome: The AIRWAYS-2 randomized clinical trial. JAMA.

[4] Wang H.E., Schmicker R.H., Daya M.R. (2018). Effect of a strategy of initial laryngeal tube insertion vs endotracheal intubation on 72-hour survival in adults with out-of-hospital cardiac arrest: A randomized clinical trial. JAMA.

[5] Watkins S., Chowdhury F.J., Norman C. (2023). Randomised trial of the clinical and cost effectiveness of a supraglottic airway device compared with tracheal intubation for in-hospital cardiac arrest (AIRWAYS-3): Protocol, design and implementation. Resusc Plus.

[6] Moskowitz A., Andrea L., Shiloh A.L. (2024). Design and implementation of the hospital airway resuscitation trial. Resusc Plus.

[7] Frerk C., Mitchell V.S., McNarry A.F. (2015). Difficult Airway Society 2015 guidelines for management of unanticipated difficult intubation in adults. Br J Anaesth.

[8] Morton S., Avery P., Kua J. (2023). Success rate of prehospital emergency front-of-neck access (FONA): a systematic review and meta-analysis. Br J Anaesth.

[9] Tricco A.C., Lillie E., Zarin W. (2018). PRISMA Extension for Scoping Reviews (PRISMA-ScR): Checklist and Explanation. Ann Intern Med.

[10] Xeropotamos N.S., Coats T.J., Wilson A.W. (1993). Prehospital surgical airway management: 1 year's experience from the Helicopter Emergency Medical Service. Injury.

[11] Warner K.J., Bulger E.M., Sharar S.R. (2009). Prehospital management of the difficult airway: A prospective cohort study. J Emerg Med.

[12] Wang H.E., Mann N.C., Jacobson K. (2011). Out-of-hospital airway management in the United States. Resuscitation.

[13] Thomas S.H., Harrison T.H., Wedel S.K. (1999). Flight crew airway management in four settings: a six-year review. Prehosp Emerg Care.

[14] Sunde G.A., Heltne J.K., Lockey D. (2015). Airway management by physician-staffed Helicopter Emergency Medical Services – a prospective, multicentre, observational study of 2,327 patients. Scand J Trauma Resusc Emerg Med.

[15] Spaite D.W., Joseph M. (1990). Prehospital cricothyrotomy: an investigation of indications, technique, complications, and patient outcome. Ann Emerg Med.

[16] Shapey I.M., Kumar D.S., Roberts K. (2012). Invasive and surgical procedures in pre-hospital care: what is the need?. Eur J Trauma Emerg Surg.

[17] Schober P., Biesheuvel T., de Leeuw M.A. (2019). Prehospital cricothyrotomies in a helicopter emergency medical service: analysis of 19,382 dispatches. BMC Emerg Med.

[18] Robinson K.J., Katz R., Jacobs L.M. (2001). A 12-year experience with prehospital cricothyrotomies. Air Med J.

[19] Prekker M.E., Carlbom D., Kwok H. (2014). The process of prehospital airway management: Challenges and solutions during paramedic endotracheal intubation. Crit Care Med.

[20] Peters J., van Wageningen B., Hendriks I. (2015). First-pass intubation success rate during rapid sequence induction of prehospital anaesthesia by physicians versus paramedics. Eur J Emerg Med.

[21] Peters J., Bruijstens L., van der Ploeg J. (2015). Indications and results of emergency surgical airways performed by a physician-staffed helicopter emergency service. Injury.

[22] Malkan R.M., Borelli C.M., Fairley R.R. (2023). Outcomes after prehospital cricothyrotomy. Med J.

[23] Kamiutsuri K., Okutani R., Kozawa S. (2013). Analysis of prehospital endotracheal intubation performed by emergency physicians: retrospective survey of a single emergency medical center in Japan. J Anesth.

[24] Jacobson L.E., Gomez G.A., Sobieray R.J. (1996). Surgical cricothyroidotomy in trauma patients: analysis of its use by paramedics in the field. J Trauma Acute Care Surg.

[25] Himmler A., McDermott C., Martucci J. (2023). Code critical airway: A collaborative solution to a catastrophic problem. Am Surg.

[26] Germann C.A., Baumann M.R., Kendall K.M. (2009). Performance of endotracheal intubation and rescue techniques by emergency services personnel in an air medical service. Prehosp Emerg Care.

[27] Gerich T.G., Schmidt U., Hubrich V. (1998). Prehospital airway management in the acutely injured patient: the role of surgical cricothyrotomy revisited. J Trauma.

[28] Gellerfors M., Fevang E., Bäckman A. (2018). Pre-hospital advanced airway management by anaesthetist and nurse anaesthetist critical care teams: a prospective observational study of 2028 pre-hospital tracheal intubations. Br J Anaesth.

[29] Diggs L.A., Yusuf J.E., De Leo G. (2014). An update on out-of-hospital airway management practices in the United States. Resuscitation.

[30] Bulger E.M., Copass M.K., Maier R.V. (2002). An analysis of advanced prehospital airway management. J Emerg Med.

[31] Brown Iii C.A., Walls R.M., Cox K. (2014). 4,871 Emergency airway encounters by air medical providers: A report of the Air Transport Emergency Airway Management (NEAR VI: “A-TEAM”) project. West J Emerg Med.

[32] Boyle M.F., Hatton D., Sheets C. (1993). Surgical cricothyrotomy performed by air ambulance flight nurses: A 5-year experience. J Emerg Med.

[33] Aziz S., Foster E., Lockey D.J. (2021). Emergency scalpel cricothyroidotomy use in a prehospital trauma service: a 20-year review. Emerg Med j.

[34] Wong E., Ng Y.-Y. (2008). The difficult airway in the emergency department. Int J Emerg.

[35] Willinge G.J.A., Hietbrink F., Leenen L.P.H. (2021). Surgical airway procedures in emergency surgical patients: Results of what has become a back-up procedure. World J Surg.

[36] Cook T, Woodall N, Frerk C (Eds). 4th National Audit Project of The Royal College of Anaesthetists and The Difficult Airway Society: Major complications of airway management in the United Kingdom: Report and findings, March 2011. 2011 (Accessed 15 April 2024 at https://www.rcoa.ac.uk/media/32521). 10.1093/bja/aer058 21447488

[37] Rosenstock C.V., Norskov A.K., Wetterslev J. (2016). Emergency surgical airway management in Denmark: a cohort study of 452 461 patients registered in the Danish Anaesthesia Database. Br J Anaesth.

[38] Okada A., Okada Y., Kandori K. (2022). Adverse events of emergency surgical front of neck airway access: an observational descriptive study. Acute Med Surg.

[39] Offenbacher J., Nikolla D.A., Carlson J.N. (2023). Incidence of rescue surgical airways after attempted orotracheal intubation in the emergency department: A National Emergency Airway Registry (NEAR) Study. Am j Emerg Med.

[40] McGill J., Clinton J.E., Ruiz E. (1982). Cricothyrotomy in the emergency department. Ann Emerg Med.

[41] Kwon Y.S., Lee C.A., Park S. (2019). Incidence and outcomes of cricothyrotomy in the “cannot intubate, cannot oxygenate” situation. Medicine.

[42] Jansen G., Scholz S.S., Rehberg S.W. (2023). Indications and measures of medical emergency teams: a retrospective evaluation of in-hospital emergency operations of the German Resuscitation Register. Minerva Anestesiol.

[43] Isaacs J.H. (2001). Emergency cricothyrotomy: long-term results. Am Surg.

[44] Gillespie M.B., Eisele D.W. (1999). Outcomes of emergency surgical airway procedures in a hospital-wide setting. The Laryngoscope.

[45] George N., Consunji G., Storkersen J. (2022). Comparison of emergency airway management techniques in the performance of emergent Cricothyrotomy. Int j Emerg.

[46] Erlandson M.J., Clinton J.E., Ruiz E. (1989). Cricothyrotomy in the emergency department revisited. J Emerg Med.

[47] DeLaurier G.A., Hawkins M.L., Treat R.C. (1990). Acute airway management. Role of cricothyroidotomy. Am Surg.

[48] Darby J.M., Halenda G., Chou C. (2018). Emergency surgical airways following activation of a difficult airway management team in hospitalized critically Ill patients: A case series. J Intensive Care Med.

[49] Cook T.M., Woodall N., Harper J. (2011). Major complications of airway management in the UK: results of the Fourth National Audit Project of the Royal College of Anaesthetists and the Difficult Airway Society. Part 2: Intensive care and emergency departments. Br J Anaesth.

[50] Beshey B.N., Helmy T.A., Asaad H.S. (2014). Emergency percutaneous tracheotomy in failed intubation. Egypt J Chest Dis Tuberc.

[51] Bair A.E., Filbin M.R., Kulkarni R.G. (2002). The failed intubation attempt in the emergency department: analysis of prevalence, rescue techniques, and personnel. J Emerg Med.

[52] Arora R.D., Rao K.N., Satpute S. (2023). Emergency tracheostomy in locally advanced anaplastic thyroid cancer. Indian J Surg Oncol.

[53] Alkhouri H., Richards C., Miers J. (2021). Case series and review of emergency front-of-neck surgical airways from The Australian and New Zealand Emergency Department Airway Registry. Emerg Med Australas.

[54] Salvino C.K., Dries D., Gamelli R. (1993). Emergency cricothyroidotomy in trauma victims. J Trauma.

[55] Paix B.R., Griggs W.M. (2012). Emergency surgical cricothyroidotomy: 24 successful cases leading to a simple 'scalpel-finger-tube' method. Emerg Med Australas.

[56] Nugent W.L., Rhee K.J., Wisner D.H. (1991). Can nurses perform surgical cricothyrotomy with acceptable success and complication rates?. Ann Emerg Med.

[57] Moroco A.E., Armen S.B., Goldenberg D. (2021). Emergency cricothyrotomy: A 10-year single institution experience. Am Surg.

[58] McIntosh S.E., Swanson E.R., Barton E.D. (2008). Cricothyrotomy in air medical transport. J Trauma.

[59] King D., Ogilvie M., Michailidou M. (2012). Fifty-four emergent cricothyroidotomies: are surgeons reluctant teachers?. Scand J Surg.

[60] Katzenell U., Lipsky A.M., Abramovich A. (2013). Prehospital intubation success rates among Israel Defense Forces providers: epidemiologic analysis and effect on doctrine. J Trauma Acute Care Surg.

[61] High K., Brywczynski J., Han J.H. (2018). Cricothyrotomy in helicopter emergency medical service transport. Air Med j.

[62] Hawkins M.L., Shapiro M.B., Cue J.I. (1995). Emergency cricothyrotomy: a reassessment. Am Surg.

[63] Graham D.B., Eastman A.L., Aldy K.N. (2011). Outcomes and long term follow-up after emergent cricothyroidotomy: is routine conversion to tracheostomy necessary?. Am Surg.

[64] Duggan L., Lockhart S., Cook T. (2018). The Airway App: exploring the role of smartphone technology to capture emergency front-of-neck airway experiences internationally. Anaesthesia.

[65] Bair A.E., Panacek E.A., Wisner D.H. (2003). Cricothyrotomy: A 5-year experience at one institution. J Emerg Med.

[66] Tobin J.M., Nordmann G.R., Kuncir E.J. (2015). Resuscitation During Critical Care Transportation in Afghanistan. J Spec Oper Med.

[67] Schauer S.G., Naylor J.F., Maddry J.K. (2018). Prehospital airway management in Iraq and Afghanistan: A descriptive analysis. South Med J.

[68] Schauer S.G., Bellamy M.A., Mabry R.L. (2015). A comparison of the incidence of cricothyrotomy in the deployed setting to the emergency department at a level 1 military trauma center: a descriptive analysis. Mil Med.

[69] Pugh H.E.J., LeClerc S., McLennan J. (2015). A review of pre-admission advanced airway management in combat casualties, Helmand Province 2013. J R Army Med Corps.

[70] Mabry R.L. (2012). An analysis of battlefield cricothyrotomy in Iraq and Afghanistan. J Spec Oper Med.

[71] Leibovici D., Gofrit O.N., Blumenfeld A. (1997). Prehospital cricothyroidotomy by physicians. Am j Emerg Med.

[72] Lairet J.R., Bebarta V.S., Burns C.J. (2012). Prehospital interventions performed in a combat zone: a prospective multicenter study of 1,003 combat wounded. J Trauma Acute Care Surg.

[73] Kyle T., le Clerc S., Thomas A. (2016). The success of battlefield surgical airway insertion in severely injured military patients: a UK perspective. J R Army Med Corps.

[74] Hudson I.L., Blackburn M.B., Staudt A.M. (2020). Analysis of casualties that underwent airway management before reaching role 2 facilities in the Afghanistan conflict 2008–2014. Mil Med.

[75] Benov A., Shkolnik I., Glassberg E. (2019). Prehospital trauma experience of the Israel defense forces on the Syrian border 2013–2017. J Trauma Acute Care Surg.

[76] Beit Ner E., Tsur A.M., Nadler R. (2021). High success rate of prehospital and en route cricothyroidotomy performed in the Israel Defense Forces: 20 years of experience. Prehosp Disaster Med.

[77] Barnard E.B.G., Ervin A.T., Mabry R.L. (2014). Prehospital and en route cricothyrotomy performed in the combat setting: a prospective, multicenter, observational study. J Spec Oper Med.

[78] Adams B.D., Cuniowski P.A., Muck A. (2008). Registry of emergency airways arriving at combat hospitals. J Trauma.

[79] Panchal A.R., Bartos J.A., Cabañas J.G. (2020). Part 3: Adult basic and advanced life support: 2020 American Heart Association guidelines for cardiopulmonary resuscitation and emergency cardiovascular care. Circulation.

[80] Spittal M.J. (1993). Evaluation of pulse oximetry during cardiopulmonary resuscitation. Anaesthesia.

[81] Aldred D., Durham M., Prokop N. (2022). Critical care paramedics' experiences of performing an emergency scalpel cricothyroidotomy: a qualitative study. Br Paramed J.

[82] Munn Z., Peters M.D., Stern C. (2018). Systematic review or scoping review? Guidance for authors when choosing between a systematic or scoping review approach. BMC Med Res Methodol.

[83] Haag A.-K., Tredese A., Bordini M. (2024). Emergency front-of-neck access in pediatric anesthesia: A narrative review. Paediatr Anaesth.

