# Supplementary material for: Emergency front-of-neck access in cardiac arrest: A scoping review
Source: Resusc Plus. 2024 May 4;18:100653. doi: 10.1016/j.resplu.2024.100653 (PMC11074978; doi:10.1016/j.resplu.2024.100653)
Supplement: Supplementary Data 1 [file mmc1.pdf]

Emergency front-of-neck access in cardiac arrest: A Scoping Review

Table of contents

S1- Completed ILCOR scoping review protocol template Page 2
S2- Data extraction items Page 7
S3- MEDLINE search strategy Page 8
S4- Embase search strategy Page 10
S5- Cochrane library search strategy Page 12

S1- Completed ILCOR scoping review protocol template

TF SR/ScR PICOST Template 16 Sept 2019 v_1.0 SAC approved 16September2019

International Liaison Committee on Resuscitation ILCOR

Task Force Based Review PICOST Template

☐ Task Force Led Systematic Review
X Task Force Led Scoping Review
☐ Need IS assistance with search strategy
X Team will use their own IS

This template is designed to help guide the preparation of a research question by Population Intervention Comparator Outcome Study design and Timeline (PICOST) to guide a Task Force based Systematic Review (TFSR) and a Task Force based Scoping Review (TFScR). This is a key step in the design of any systematic review (SR) or scoping review. The initial PICOST for a Systematic Review is typically very specific but that for a Scoping review may be very broad. The TF based Review PICOST template is to be prepared by the Task Force SR/ScR team lead with the oversight of the Task Force Scientific Advisory Committee (SAC) representative(s), and then forwarded to SAC for acknowledgement.
The Task Force SAC representative is the key liaison between SAC and the Taskforce.

PICOST Short Title *(edit)*	**Front of neck access in cardiac arrest**

1. TF Research Question based on PICOST

(Population, Intervention, Control, Outcomes, Study design and Timeframe)

**PICOST**	**Description** *(with recommended text)*
**Population**	Adult patients in cardiac arrest in any setting, where adequate ventilation cannot be rapidly achieved using basic/ advanced airway management strategies
**Intervention**	Front-of-neck access attempt
**Comparison**	Ongoing attempts at basic/advancedairway management strategies.
**Outcomes**	Any clinical outcome. (preset text)
**Study Design**	Randomized controlled trials (RCTs) and non-randomized studies (non-randomized controlled trials, interrupted time series, controlled before-and-after studies, cohort studies), case series (cases ≥ *5)* are eligible for inclusion. Unpublished studies (e.g., conference abstracts, trial protocols) are excluded.
**Timeframe**	All years and all languages are included as long as there is an English abstract

2. Task Force Systematic/Scoping Review (TFSR/TFScR) Team

1

TF SR/ScR PICOST Template 16 Sept 2019 v_1.0 SAC approved 16September2019

***All TFSR/TFScR team members (including non-TF members) are expected to have completed the generic AHA COI documentation.**
TF chair or delegate will confirm COI through topic specific disclosures prior to assignment.
ILCOR COI Policy and the COI Committee are resources to address any questions.

- ☐ **TFC attestation: I have checked for fiscal and intellectual conflict of interests and found none**
- ☐ **I have check for fiscal and intellectual conflict of interests; Author XXX (eg-has published study on Y and is excluded from bias assessment and xxxxxxxx**

TFSR/TFScR team

Role	Name	Topic specific COI reviewed	Notes
*Task Force SR/ScR Team Lead	Keith Couper		(needs to be TF member*)
* Task Force SR/ScR Content Experts			Can be from outside of TF
*TF SAC representative(s)			(assigned by SAC)
Research fellows	Mohammed Aljanoubi<br>Abdulkarim Almazrua		

Notes on COI: e.g. Author XXX has published study on Y and is excluded from bias assessment and xxxxxxxx

3. Pre-existing PICOs Related to scope of work for this PICOST (e.g. 2010, 2015):

Insert all PICOs as worded on the master document and include AHA number to assist in retrieval of previous work. (note: This information is available in the file: ILCOR PICO List on ilcor.org). Insert Not Applicable if none exist.

Not Applicable

4. Definitions: *(This should include definitions of all the key relevant terms identified in the PICOST and in the body of literature related to this topic)*

Adequate ventilation: ventilation that is, in the view of the attending healthcare provider, adequate for current treatment delivery

Basic/advanced airway management strategies: Conventional strategies including head positioning, use of simple airway adjuncts, supraglottic airway, and tracheal intubation (e.g. Newell 2018 190; https://doi.org/10.1186/s13054-018-2121-y)

Front-of-neck access: access to the trachea through the front of the neck (e.g. needle cricothyroidotomy, tracheostomy) to facilitate ventilation/ oxygenation

5. **Notes:** Please add any relevant nuances and subtleties of the task force discussion; including anything that doesn't fit in any other PICOST section but the task force/CEs feels this information is contributory to the question: e.g. pre-specified subgroups. **If it is anticipated that there will be insufficient direct evidence, and**

indirect evidence will be used to answer the question, it needs to be documented clearly what is meant by indirect and confirm indirect evidence exists.

The area is understudied.
Our initial review of the literature indicates that we are unlikely to identify any evidence that directly addresses our PICOST.

If direct evidence cannot be identified, we plan to summarise evidence across four key areas:
- Frequency of front-of-neck access attempts across different populations (e.g. cardiac arrest, trauma)
- Reported success rates of front-of-neck access attempts (including details of population, type of technique)
- Complications associated with front-of-neck access attempts (including details of population, type of technique)
- Outcomes of patients that receive front-of-neck access attempts (including details of population, type of technique)

6. **Possible Outcomes:** *(These may be updated/modified after the SR search is performed and the total number of critical or important outcomes should be no more than 7)*

For direct evidence:
- Health-related quality of life
- Survival to hospital discharge/ 30 days with favorable neurological function
- Survival to hospital discharge/ 30 days
- Return of spontaneous circulation

For indirect evidence (as outlined in section 5):
- Frequency of front-of-neck access attempts
- Success rates
- Complications

7. ***Key recent studies:*** *(To guide this and future searches, please include sentinel papers or any anticipated publications (including estimated date of publication) that are appropriate to answer this PICOST. Please insert full references)*

Aziz S, Foster E, Lockey DJ, et al. Emergency scalpel cricothyroidotomy use in a prehospital trauma service: a 20-year review. Emergency Medicine Journal 2021;38:349-354.

Murphy D, Bulger N, Harrington B, et al. Fewer Tracheal Intubation Attempts are Associated with Improved Neurologically Intact Survival Following Out-of-Hospital Cardiac Arrest. Resuscitation 2021; https://doi.org/10.1016/j.resuscitation.2021.07.001

8. **Is there an existing detailed (e.g. IS developed) search strategy?**

Yes ☒ or No ☐

If no, please complete the following questions to provide assistance to the information specialist.

Search strategy in question 10 was developed in conjunction with information specialist

9. ***Recent systematic reviews:*** *(directly or indirectly addressing this PICO. Please insert full references)*

DeVore EK, Redmann A, Howell R, Khosla S. Best practices for emergency surgical airway: A systematic review. Laryngoscope Investig Otolaryngol. 2019;4(6):602-608. doi: 10.1002/lio2.314.

Langvad S, Hyldmo PK, Nakstad AR, Vist GE, Sandberg M. Emergency cricothyrotomy--a systematic review. Scand J Trauma Resusc Emerg Med. 2013;21:43. doi: 10.1186/1757-7241-21-43.

Park L, Zeng I, Brainard A. Systematic review and meta-analysis of first-pass success rates in emergency department intubation: Creating a benchmark for emergency airway care. Emerg Med Australas. 2017 Feb;29(1):40-47. doi: 10.1111/1742-6723.12704.

Zasso FB, You-Ten KE, Ryu M, Losyeva K, Tanwani J, Siddiqui N. Complications of cricothyroidotomy versus tracheostomy in emergency surgical airway management: a systematic review. BMC Anesthesiol. 2020;20(1):216. doi: 10.1186/s12871-020-01135-2.

10. Suggested specific search terms/keywords

1	Tracheostom*.mp. or exp Tracheostomy/
2	Tracheotom*.mp. or exp Tracheotomy/
3	cricothy*.mp.
4	front of neck.mp.
5	High Frequency Jet Ventilation.mp. or exp High-Frequency Jet Ventilation/
6	HFJV.mp.
7	Surgical airway.mp.
8	1 or 2 or 3 or 4 or 5 or 6 or 7
9	exp Heart Arrest/
10	(cardiac arrest* or CPR).mp. or exp Cardiopulmonary Resuscitation/
11	Resuscitation*.mp.
12	exp Advanced Cardiac Life Support/ or Advanced Cardiac Life Support*.mp.
13	exp Tachycardia, Ventricular/ or exp Ventricular Fibrillation/ or VF.mp.
14	exp Multiple Trauma/
15	polytrauma.mp.
16	Out of hospital.mp.
17	Prehospital.mp.
18	Pre-hospital.mp.
19	Emergency medical services.mp. or exp Emergency Medical Services/
20	exp Emergency Responders/
21	Emergency medical technician.mp. or exp Emergency Medical Technicians/
22	EMT.mp.
23	Paramedic*.mp.
24	exp Ambulances/
25	exp Air Ambulances/ or Air Ambulance*.mp.
26	Helicopter emergency medical services.mp.
27	HEMS.mp.
28	Emergency department.mp. or exp Emergency Service, Hospital/
29	Emergency room.mp.

30	exp Emergency Treatment/
31	exp Emergency Medicine/
32	exp Hospital Rapid Response Team/
33	exp Military Health Services/ or exp Hospitals, Military/ or Military*.mp.
34	9 or 10 or 11 or 12 or 13 or 14 or 15 or 16 or 17 or 18 or 19 or 20 or 21 or 22 or 23 or 24 or 25 or 26 or 27 or 28 or 29 or 30 or 31 or 32 or 33
35	Operat* room.mp. or exp Anesthesia/
36	intensive care unit*.mp.
37	exp Critical Care/ or Critical care unit*.mp.
38	Operat* theatre.mp. [mp=title, abstract, original title, name of substance word, subject heading word, floating sub-heading word, keyword heading word, organism supplementary concept word, protocol supplementary concept word, rare disease supplementary concept word, unique identifier, synonyms]
39	35 or 36 or 37 or 38
40	Can* intubate can* oxygenate*.mp.
41	CICO.mp.
42	Can* intubate can* Ventilate*.mp.
43	CICV.mp.
44	Difficult intubation.mp.
45	Failed intubation.mp.
46	Difficult airway.mp.
47	40 or 41 or 42 or 43 or 44 or 45 or 46
48	39 and 47
49	34 or 48
50	8 and 49

S2- Data extraction items

Data Extractor	Paper Characteristics	Direct or Indirect Outcome
Name	Paper title	Short-term survival: Includes survival to hospital admission and survival in hospital
Email	First author's name	Medium-term survival: Survival to hospital discharge
Extraction date	Year of publication	Long-term survival: Survival for 30 days or discharge with favourable neurological outcome
	Country of the data	Return of spontaneous circulation
	Research period	Frequency of FONA attempts
	Population studies	Success rate
	Study design	Complications
	Number of participants	
	Participant description	
	Patients' characteristics	
	Provider of eFONA	
	Setting of eFONA	
	Population studied	
	Indications for eFONA	

S3- MEDLINE search strategy

1 Tracheostom*.mp. or exp Tracheostomy/
2 Tracheotom*.mp. or exp Tracheotomy/
3 cricothy*.mp.
4 front of neck.mp.
5 High Frequency Jet Ventilation.mp. or exp High-Frequency Jet Ventilation /
6 HFJV.mp.
7 Surgical airway.mp.
8 1 or 2 or 3 or 4 or 5 or 6 or 7
9 exp Heart Arrest/
10 (cardiac arrest* or CPR).mp. or exp Cardiopulmonary Resuscitation/
11 Resuscitation*.mp.
12 exp Advanced Cardiac Life Support/ or Advanced Cardiac Life Support*.mp.`
13 exp Tachycardia, Ventricular/ or exp Ventricular Fibrillation/ or VF.mp.
14 exp Multiple Trauma/
15 polytrauma.mp.
16 Out of hospital.mp.
17 Prehospital.mp.
18 Pre-hospital.mp.
19 Emergency medical services.mp. or exp Emergency Medical Services/
20 exp Emergency Responders/
21 Emergency medical technician.mp. or exp Emergency Medical Technicians/
22 EMT.mp.
23 Paramedic*.mp.
24 exp Ambulances/
25 exp Air Ambulances/ or Air Ambulance*.mp.
26 Helicopter emergency medical services.mp.
27 HEMS.mp.
28 Emergency department.mp. or exp Emergency Service, Hospital/
29 Emergency room.mp.
30 exp Emergency Treatment/
31 exp Emergency Medicine/
32 exp Hospital Rapid Response Team/
33 exp Military Health Services/ or exp Hospitals, Military/ or Military*.mp.
34 9 or 10 or 11 or 12 or 13 or 14 or 15 or 16 or 17 or 18 or 19 or 20 or 21 or 22 or 23 or 24 or 25 or 26 or 27 or 28 or 29 or 30 or 31 or 32 or 33
35 Operat* room.mp. or exp Anesthesia/
36 intensive care unit*.mp.
37 exp Critical Care/ or Critical care unit*.mp.
38 Operat* theatre.mp. [mp=title, abstract, original title, name of substance word, subject heading word, floating sub-heading word, keyword heading word, organism supplementary concept word, protocol supplementary concept word, rare disease supplementary concept word, unique identifier, synyms]
39 35 or 36 or 37 or 38
40 Can* intubate can* oxygenate*.mp.
41 CICO.mp.
42 Can* intubate can* Ventilate*.mp.
43 CICV.mp.
44 Difficult intubation.mp.
45 Failed intubation.mp.
46 Difficult airway.mp.
47 40 or 41 or 42 or 43 or 44 or 45 or 46

48	39 and 47
49	34 or 48
50	8 and 49

S4- Embase search strategy

1 Tracheostom*.mp. or exp Tracheostomy/
2 Tracheotom*.mp. or exp Tracheotomy/
3 cricothy*.mp.
4 front of neck.mp.
5 High Frequency Jet Ventilation.mp. or exp High-Frequency Jet Ventilation/
6 HFJV.mp.
7 Surgical airway.mp.
8 1 or 2 or 3 or 4 or 5 or 6 or 7
9 exp Heart Arrest/
10 (cardiac arrest* or CPR).mp. or exp Cardiopulmonary Resuscitation/
11 Resuscitation*.mp.
12 exp Advanced Cardiac Life Support/ or Advanced Cardiac Life Support*.mp.
13 exp Tachycardia, Ventricular/ or exp Ventricular Fibrillation/ or VF.mp.
14 exp Multiple Trauma/
15 polytrauma.mp.
16 Out of hospital.mp.
17 Prehospital.mp.
18 Pre-hospital.mp.
19 Emergency medical services.mp. or exp Emergency Medical Services/
20 exp Emergency Responders/
21 Emergency medical technician.mp. or exp Emergency Medical Technicians/
22 EMT.mp.
23 Paramedic*.mp.
24 exp Ambulances/
25 exp Air Ambulances/ or Air Ambulance*.mp.
26 Helicopter emergency medical services.mp.
27 HEMS.mp.
28 Emergency department.mp. or exp Emergency Service, Hospital/
29 Emergency room.mp.
30 exp Emergency Treatment/
31 exp Emergency Medicine/
32 exp Hospital Rapid Response Team/
33 exp Military Health Services/ or exp Hospitals, Military/ or Military*.mp.
34 9 or 10 or 11 or 12 or 13 or 14 or 15 or 16 or 17 or 18 or 19 or 20 or 21 or 22 or 23 or 24 or 25 or 26 or 27 or 28 or 29 or 30 or 31 or 32 or 33
35 Operat* room.mp. or exp Anesthesia/
36 intensive care unit*.mp.
37 exp Critical Care/ or Critical care unit*.mp.
38 Operat* theatre.mp. [mp=title, abstract, original title, name of substance word, subject heading word, floating sub-heading word, keyword heading word, organism supplementary concept word, protocol supplementary concept word, rare disease supplementary concept word, unique identifier, synyms]
39 35 or 36 or 37 or 38
40 Can* intubate can* oxygenate*.mp.
41 CICO.mp.
42 Can* intubate can* Ventilate*.mp.
43 CICV.mp.
44 Difficult intubation.mp.
45 Failed intubation.mp.
46 Difficult airway.mp.
47 40 or 41 or 42 or 43 or 44 or 45 or 46

48	39 and 47
49	34 or 48
50	8 and 49

S5- Cochrane library search strategy

1 Tracheostom*
2 MeSH descriptor: Tracheostomy] explode all trees
3 Tracheotom*
4 MeSH descriptor: [Tracheotomy] explode all trees
5 cricothy*
6 front of neck
7 MeSH descriptor: [High-Frequency Jet Ventilation] explode all trees
8 High Frequency Jet Ventilation
9 HFJV
10 Surgical airway
11 #1 or #2 or #3 or #4 or #5 or #6 or #7 or #8 or #9 or #10
12 MeSH descriptor: [Heart Arrest] explode all trees
13 cardiac arrest* or CPR
14 MeSH descriptor: [Cardiopulmonary Resuscitation] explode all trees
15 MeSH descriptor: [Advanced Cardiac Life Support] explode all trees
16 Advanced Cardiac Life Support*
17 MeSH descriptor: [Tachycardia, Ventricular] explode all trees
18 MeSH descriptor: [Ventricular Fibrillation] explode all trees
19 VF.
20 MeSH descriptor: [Multiple Trauma] explode all trees
21 polytrauma
22 Out of hospital
23 Prehospital
24 Pre-hospital
25 Emergency medical services
26 MeSH descriptor: [Emergency Medical Services] explode all trees
27 MeSH descriptor: [Emergency Responders] explode all trees
28 Emergency medical technician
29 MeSH descriptor: [Emergency Medical Technicians] explode all trees
30 EMT
31 Paramedic*
32 MeSH descriptor: [Ambulances] in all MeSH products
33 MeSH descriptor: [Air Ambulances] explode all trees
34 Air Ambulance*
35 Helicopter emergency medical services
36 HEMS
37 Emergency department
38 MeSH descriptor: [Emergency Service, Hospital] explode all trees
39 Emergency room
40 MeSH descriptor: [Emergency Treatment] explode all trees
41 MeSH descriptor: [Emergency Medicine] explode all trees
42 MeSH descriptor: [Hospital Rapid Response Team] explode all trees
43 MeSH descriptor: [Military Health Services] explode all trees
44 MeSH descriptor: [Hospitals, Military] explode all trees
45 Military*
46 #12 or #13 or #14 or #15 or #16 or #17 or #18 or #19 or #20 or #21 or #22 or #23 or #24 or #25 or #26 or #27 or #28 or #29 or #30 or #31 or #32 or #33 or #34 or #35 or #36 or #37 or #38 or #39 or #40 or #41 or #42 or #43 or #44 or #45 or #46
47 Operat* room
48 MeSH descriptor: [Anesthesia] explode all trees

49	intensive care unit*
50	MeSH descriptor: [Critical Care] explode all trees
51	Critical care unit*
52	Operat* theatre
53	#48 or #49 or #50 or #51 or #52 or #53
54	Can* intubate can* oxygenate*
55	CICO
56	Can* intubate can* Ventilate*
57	CICV
58	Difficult intubation
59	Failed intubation
60	Difficult airway
61	#55 or #56 or #57 or #58 or #59 or #60 or #61
62	#54 and #62
63	#47 or # 63
64	#11 and #64
